# Supplementary material for: Does Participation in Sports Influence the Prevalence of and Initiation into Multiple Substance Misuse in Adolescence? A Two-Year Prospective Analysis
Source: Children (Basel). 2020 Aug 22;7(9):109. doi: 10.3390/children7090109 (PMC7552612; doi:10.3390/children7090109)
Supplement: Supplementary file 1 [file children-07-00109-s001.pdf]

children

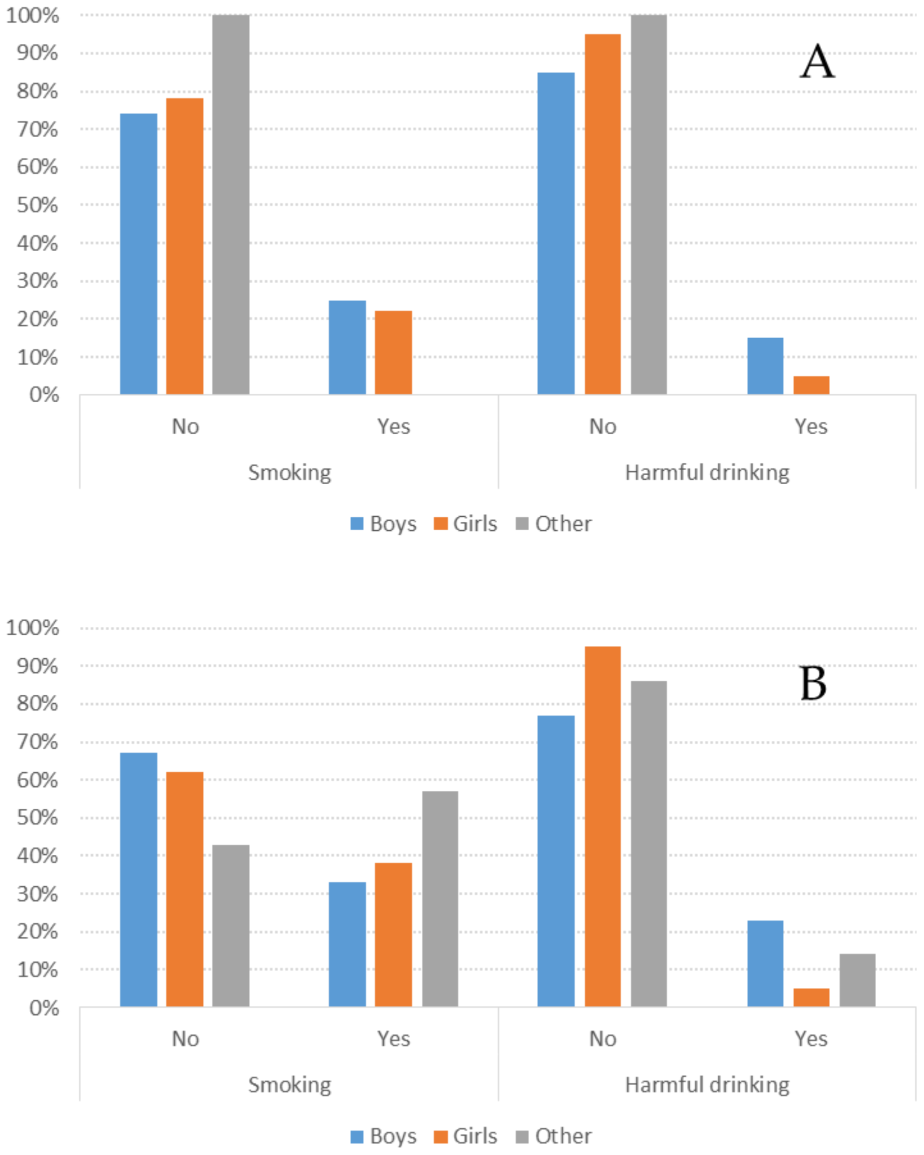


**Figure S1.** Prevalence of smoking and harmful drinking in studied adolescents from southern Croatia, at baseline (A), and follow-up (B), according to gender.
